# Death from Inhalation of Chloroform in Lisbon

**Published:** 1860-06

**Authors:** 


*Death from Inhalation of Chloroform in Lisbon.*—On the 12th of February, a man of thirty was to have been operated upon, at the St. Joseph Hospital of Lisbon, for a cystic tumor of the face. Chloroform was administered by means of a piece of lint, covered with a thin strip of linen, and held at a short distance from the nose and mouth. The stage of excitement suddenly ceased, the patient turned pale and pulseless, and made a long inspiration. He drew in long breaths at more and more distant intervals until he died—just six minutes after the chloroform had been placed against his mouth. The actual contact of the lint moistened with the chloroform had hardly lasted two minutes. On a *post mortem* examination, the lungs were found gorged with blood and ecchymosed, the right auriculo-ventricular orifice dilated, the aorta full of brown, fluid blood, the cerebral matter and meninges injected.—*Gaz. Med. de Lyon et O Archivo Universal de Lisboa.*

